# Increased GIRK channel activity prevents arrhythmia in mice with heart failure by enhancing ventricular repolarization

**DOI:** 10.1038/s41598-023-50088-2

**Published:** 2023-12-18

**Authors:** Xue An, Hana Cho

**Affiliations:** 1 Department of Physiology, Sungkyunkwan University School of Medicine, Suwon, 16419 Korea; 2 Present Address: Department of Critical Care Medicine, Renji Hospital, School of Medicine, Shanghai Jiaotong University, Shanghai, China

**Keywords:** Physiology, Cardiology

## Abstract

Ventricular arrhythmia causing sudden cardiac death is the leading mode of death in patients with heart failure. Yet, the mechanisms that prevent ventricular arrhythmias in heart failure are not well characterized. Using a mouse model of heart failure created by transverse aorta constriction, we show that GIRK channel, an important regulator of cardiac action potentials, is constitutively active in failing ventricles in contrast to normal cells. Evidence is presented indicating that the tonic activation of M_2_ muscarinic acetylcholine receptors by endogenously released acetylcholine contributes to the constitutive GIRK activity. This constitutive GIRK activity prevents the action potential prolongation in heart failure ventricles. Consistently, GIRK channel blockade with tertiapin-Q induces QT interval prolongation and increases the incidence of arrhythmia in heart failure, but not in control mice. These results suggest that constitutive GIRK channels comprise a key mechanism to protect against arrhythmia by providing repolarizing currents in heart failure ventricles.

## Introduction

G protein-gated inwardly-rectifying potassium (GIRK) channels play a role in the control of cellular excitability in brain and heart in response to various neurotransmitters, such as acetylcholine (ACh), dopamine, opioids, serotonin, somatostatin, adenosine, and GABA, which target pertussis toxin (PTX)-sensitive (G_i_ and G_o_) G protein-coupled receptors (GPCRs). GPCR activation then leads to the dissociation of G_βγ_ subunits from the heterotrimeric G protein complex and the activation of GIRK channels via the binding of G_βγ_ to the channel^1^. Abundant in the pacemaker cells in the sino-atrial (SA) node, where they were originally discovered^2^, GIRK channels are stimulated by M_2_ muscarinic acetylcholine receptor (mAChR) activation in response to ACh released upon parasympathetic stimulation. Under physiological conditions, activation of GIRK channels hyperpolarize SA nodal cells and thus slow the heart rate^3^. The cardiac GIRK channel, often referred to as ACh-regulated K^+^ current (I_KACh_), is a heterotetramer consisting of GIRK1 and GIRK4 subunits^1^. Pharmacological investigations of GIRK channels and studies in animal models have shown that these channels are also important in the control of the excitability of atrio-ventricular (AV) node and atrial myocytes where they hyperpolarize the membrane potential and hasten action potential (AP) repolarization, shortening AP duration (APD) upon parasympathetic stimulation^4–7^. Thus, cardiac GIRK channels are key mediators of the parasympathetic regulation of heart functions. In contrast to nodal and atrial cells, in ventricles, ACh-induced activation of GIRK channels is known to be weak^8,9^, and parasympathetic innervation is sparse.

Heart failure is a leading cause of morbidity and mortality worldwide. Despite remarkable improvements in medical therapy, the prognosis of patients with heart failure is morbid, with about 50% of patients dying within 5 years after the initial diagnosis, which exceeds most types of cancer^10^. Remarkably, of the deaths in patients with heart failure, approximately half are sudden and unexpected deaths caused by ventricular arrhythmia^11^. Heart failure induces an alteration in the functional activity of key ion channels and transporters in ventricular myocytes; this electrophysiological remodeling is known to enhance the propensity for arrhythmia^12,13^. However, distinct ion channel remodeling can be protective or maladaptive in the development of arrhythmia. With such complexities involved in arrhythmias, pharmacological therapy for ventricular arrhythmia in heart failure patients remains highly inadequate, often exacerbating symptoms and heightening the risk of sudden death^12^. Therefore, development of therapeutic strategies requires a detailed understanding of heart failure-induced changes in ion channel function.

Atrial fibrillation increases the constitutive form of GIRK channel activity as a consequence of atrial remodeling, which might exert proarrhythmic effects^14,15^. Constitutive GIRK channel activity refers to the GIRK channel activity generated independent of neural stimulation. Several mechanisms may contribute to the development of constitutively active GIRK currents^16^. For example, the tonic activation of GABAA receptors by ambient extracellular GABA contributes to the constitutive GIRK channel activity in dentate granule cells of the hippocampus^17^. Alternatively, receptor-independent GIRK activation might be involved in constitutive GIRK activity, including increased availability of G_βγ_-subunits, and alterations and phosphorylation of the GIRK channel composition^16^. Since they are constitutively active at resting membrane potential, constitutive GIRK channels can constitute basal inward rectifier K^+^ currents, setting the low resting membrane potential and serving as a repolarizing current. This was supported by the observation that constitutive GIRKs play a key role in characteristic low resting membrane potential and low excitability of mature dentate granule cells in the hippocampus^18^. Despite their role in membrane excitability, however, whether such constitutive activity of GIRK channels develops in heart failure ventricles and their contribution to lethal ventricular arrhythmia remain unknown.

In the present study, we uncovered that constitutive GIRK channel activity is induced in ventricular myocytes from a heart failure mouse model created by transverse aorta constriction (TAC). Further activation of GIRK activity by exogenous ACh is also higher in TAC mice than in sham mice. A functional study showed that constitutive GIRK activity plays a key role in the ventricular repolarization in TAC mice. Endogenously released ACh from failing ventricular myocytes seems to be responsible for constitutive GIRK activity, likely through tonic activation of M_2_ mAChR receptors, since blocking of the M_2_ mAChR signaling or ACh synthesis inhibits constitutive GIRK activity. Consistent with a major role of constitutive GIRK in the repolarization of TAC ventricular myocytes, electrocardiography (ECG) analysis showed that GIRK channel blockade with tertiapin-Q markedly prolonged the QT interval and increased the incidence of ventricular arrhythmia in TAC mice while having no significant effect in sham mice. Together, these data suggest that GIRK channel activity is increased in heart failure mouse ventricular myocytes, which limits excessive APs and QT prolongation, providing new insights into GIRK channels as possible targets for improving ventricular function and reducing arrhythmias in heart failure.

## Results

### Constitutive GIRK activity is enhanced in TAC ventricular myocytes

**Figure 1:**
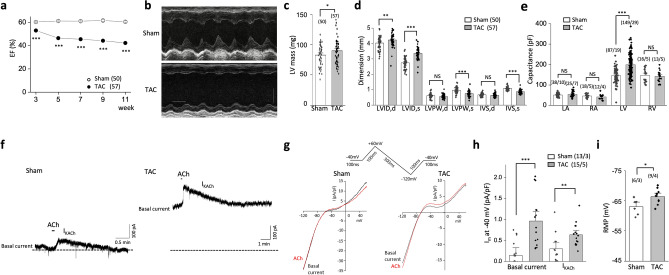
Basal and acetylcholine (ACh)-induced inward rectifier currents are increased in left ventricular myocytes from transverse aorta constriction (TAC) mice. Mice were subjected to the TAC (n = 57) or sham (n = 50) operation, as described in the Methods. (**a**) The temporal changes in ejection fraction (EF) over 11 weeks after surgery in sham and TAC mice. In TAC mice, the EF decreased gradually. (**b**) Representative M-mode echocardiographic images of the left ventricle (LV), time stamp: 100 ms, vertical bar: 2 mm. (**c**,**d**) Average values for the LV mass (**c**) and the indicated echocardiographic parameter (**d**) from sham and TAC mice at 11 weeks after surgery. TAC mice showed clear LV structural abnormalities compared with age-matched sham-operated mice. LVID;d or LVID;s the internal dimension of LV; diastolic or systolic, LVPW;d or LVPW;s the postwall thickness of LV; diastolic or systolic, IVS;d or IVS;s the interventricular septum; diastolic or systolic. (**e**) Capacitance of myocytes from the left atrium (LA), right atrium (RA), LV, and right ventricle (RV) of sham and TAC mice. (**f**) Representative current trace of membrane currents in LV myocytes of sham and TAC mice at a holding potential of − 40 mV. ACh (10 µM) was applied during the periods indicated. The dotted line indicates zero current level. (**g**) Current–voltage relationship under basal conditions (basal current) and after ACh treatment in response to a ramp pulse from − 120 to + 60 mV (at a speed of ± 0.6Vs^-1^) from the holding potential of − 40 mV. (**h**,**i**) Summary data for basal current and ACh-activated GIRK current density (**h**) and resting membrane potential (RMP) (**i**) in sham and TAC. Data represent means ± SEM. The numbers indicate number of myocytes/mice. NS indicates not significantly different. *P < 0.05, **P < 0.01, ***P < 0.001, Student’s t-test.

To characterize the potential role of GIRK channels during heart failure, we measured whole-cell currents in a murine model of TAC-induced heart failure. Adult mice were subjected to TAC (n = 57) or were sham-operated (n = 50) and were monitored biweekly by echocardiography and ECG. In TAC mice, the ejection fraction decreased gradually, and they showed clear left ventricular (LV) dysfunction and structural abnormalities compared with age-matched sham-operated mice, as assessed by echocardiographic parameters after 11 weeks (Fig. 1a–d). Consistent with LV dysfunction, the capacitance of LV myocytes 11 weeks after TAC significantly increased when compared with the sham (189.6 ± 4.5 pF, n = 149/29 [myocytes/mice] vs. 147.4 ± 4.8 pA/pF, n = 87/19; *P* < 0.001), while not that of the right ventricular myocytes nor that of the atrial myocytes increased (Fig. 1e). At the 11-week endpoint, we recorded the membrane currents of TAC and sham LV myocytes under basal conditions (basal current) and in response to 10 μM ACh (Fig. 1f,g). The ACh-sensitive current component of sham and TAC LV myocytes were obtained by subtracting the basal current from the current–voltage (I-V) curves in the presence of ACh, as shown in Supplemental Fig. 1. Whereas ACh-sensitive current components of sham LV myocytes didn’t show the level of rectification for GIRK channels, those of TAC LV myocytes showed a typical inward rectification known for GIRK currents with a reversal potential of around − 70 mV, which is close to the equilibrium potential of the potassium ion as calculated using the Nernst equation^19^, indicating that the increase in current amplitude during exposure to ACh results from the activation of GIRK currents. TAC LV myocytes showed a larger basal current compared to sham LV myocytes. Absolute values of the basal current at a holding potential (V_h_) of − 40 mV were 0.1 ± 0.1 pA/pF (n = 13/3 [myocytes/mice]) in sham mice and 0.9 ± 0.2 pA/pF (n = 15/5) in TAC mice (*P* < 0.001; Fig. 1h). Compared to sham LV myocytes, TAC LV myocytes demonstrated an increased response to 10 μM ACh with an increased peak density of the outward current (I_KACh_) (Fig. 1h). Consistent with increases in the basal inward rectifier K^+^ currents, the resting membrane potential was hyperpolarized in TAC LV myocytes compared to sham LV myocytes (Fig. 1i). These data suggest that ACh-induced GIRK activation and the basal inward rectifier K^+^ current were increased in LV myocytes from a mouse model of heart failure.

**Figure 2:**
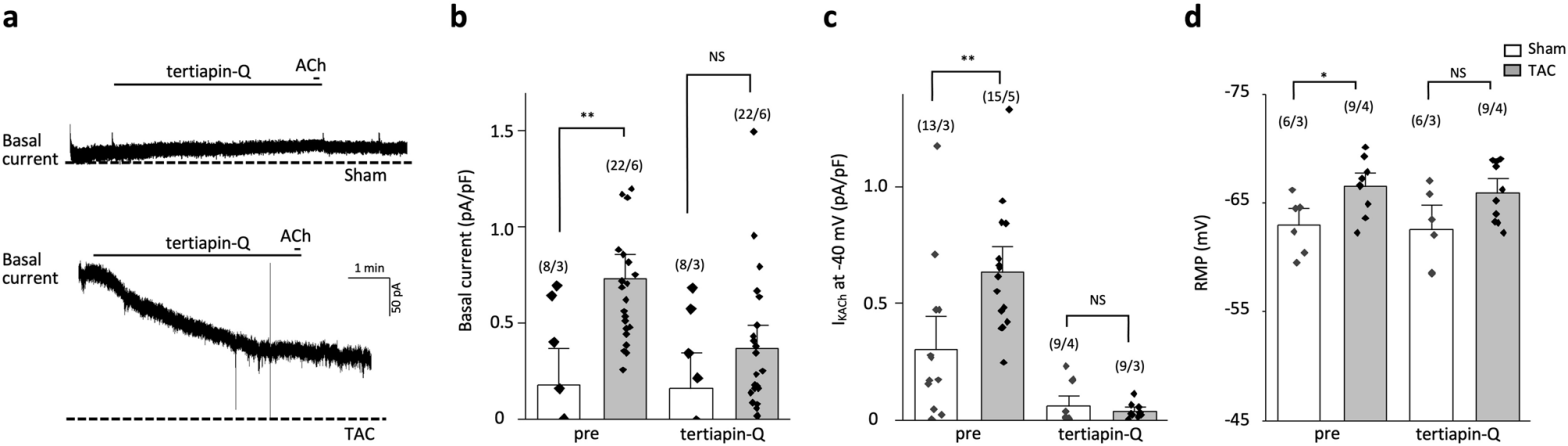
Constitutive GIRK current is increased in left ventricular myocytes from transverse aorta constriction (TAC) mice. (**a**) Tertiapin-Q (300 nM) was applied before acetylcholine (ACh; 10 μM) treatment in left ventricular (LV) myocytes from sham (upper panel) and TAC (lower panel) mice. Currents were recorded at a holding potential of − 40 mV. (**b**–**d**) Summarized data for the effects of 300 nM tertiapin-Q on basal current density (**b**), ACh-activated GIRK current density (**c**), and resting membrane potential (RMP) (**d**) in sham and TAC LV myocytes. The numbers indicate number of myocytes/mice. NS indicates not significantly different. *P < 0.05, **P < 0.01, Student’s t-test.

Knowing that a constitutive GIRK current contributes to the higher basal inward rectifier K^+^ current in atrial myocytes from chronic atrial fibrillation^14^, we tested whether constitutive GIRK activity might contribute to the enhanced basal current in TAC mice. The selective GIRK channel blocker tertiapin-Q^20^ was used to discriminate between the contribution of the GIRK current and the background inward rectifier K^+^ current I_K1_ in the enhanced basal current in TAC mice. Tertiapin-Q treatment (300 nM) reduced the basal current in TAC LV myocytes, abolishing the difference in basal currents between sham and TAC mice (sham LV: 0.2 ± 0.1 pA/pF (n = 8/3); TAC LV, 0.4 ± 0.1 pA/pF (n = 22/6); *P* = 0.16; Fig. 2a,b). These results point to GIRK currents, but not I_K1_, as a potential cause of the enhanced basal currents in TAC LV myocytes. Consistent with previous reports^14,21^, the application of 300 nM tertiapin-Q during ACh application reduced the ACh-induced GIRK activities in both sham and TAC LV myocytes (Fig. 2a,c). Notably, tertiapin-Q (300 nM) also abolished the difference in the resting membrane potential between sham and TAC LV myocytes (Fig. 2d). Together, these results suggest that heart failure increases constitutive GIRK channel activity in ventricular myocytes.

### Constitutively active GIRK channels are required for ventricular repolarization in failing myocytes

**Figure 3:**
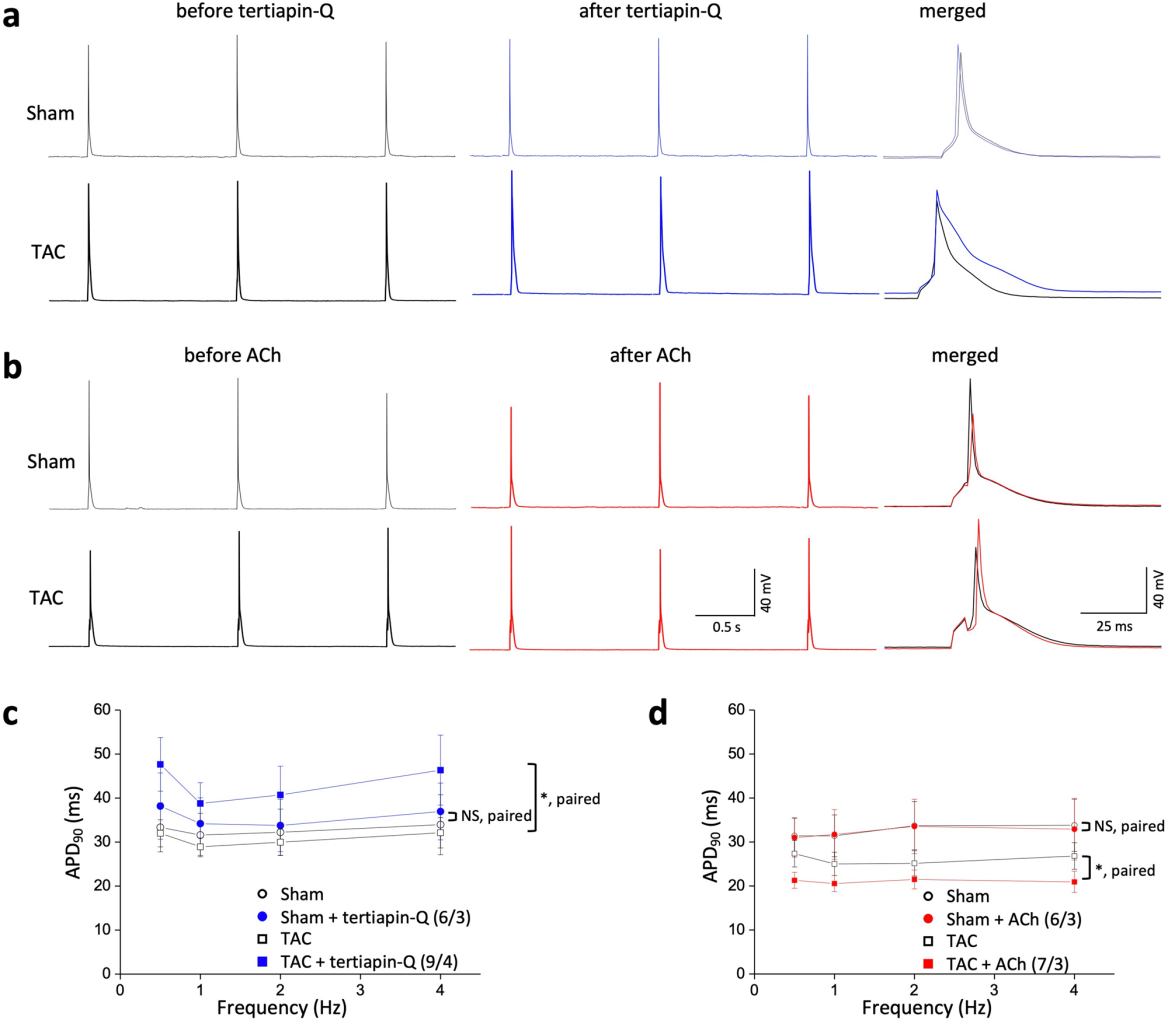
Transverse aorta constriction (TAC) ventricular myocytes exhibit enhanced susceptibility of tertiapin-Q and acetylcholine (ACh)-induced action potential (AP) modulation. (**a**,**b**) Representative AP traces in left ventricular (LV) myocytes stimulated at 1 Hz from sham (upper panel) and TAC (lower panel) before (black) and after 300 nM tertiapin-Q (**a**, blue) or 10 μM ACh treatment (**b**, red). Right, superimposed AP traces measured before and after exposure of tertiapin-Q (**a**) or ACh (**b**). Tertiapin-Q or ACh altered action potential duration (APD) only in TAC myocytes. (**c**,**d**) Summary data of APD at 90% repolarization (APD_90_) in LV myocytes from sham and TAC stimulated at 0.5 Hz, 1 Hz, 2 Hz, and 4 Hz in response to tertiapin-Q or ACh treatment. Data represent means ± SEM. The numbers indicate number of myocytes/mice. NS indicates not significantly different. *P < 0.05, Student’s paired or unpaired t-test was used as indicated.

The small outward K^+^ current through GIRK channels has a profound impact on repolarization of cardiac AP^22,23^. To examine whether enhanced GIRK activity contributes to ventricular repolarization, we tested the effects of tertiapin-Q on ventricular APs in sham and TAC LV myocytes. APs were elicited in the whole-cell configuration of the patch clamp by applying depolarizing current pulses over a range of stimulation frequencies. Figure 3a shows typical APs recorded at 1 Hz in a LV myocytes from sham and TAC mice before (left) and after (right) tertiapin-Q treatment (300 nM, 5 min). AP characteristics of LV myocytes prior to the administration of any drugs were similar in the sham and TAC mice. No statistically significant differences were observed between the two groups in AP amplitude, AP duration at 90% repolarization (APD_90_), or V_max_ (Supplemental Fig. 2). Consistent with the tertiapin-sensitive constitutive component of GIRK activity, tertiapin-Q induced a prolongation of APD_90_ at all stimulation rates in TAC LV myocytes, as summarized in Fig. 3c. For example, at a stimulation rate of 1 Hz, the APD_90_ of TAC LV myocytes increased by 34% after tertiapin-Q administration, to 38.8 ± 4.7 ms (n = 9/4, *P* < 0.05). In contrast, tertiapin-Q had no significant effect on AP morphology and APD_90_ in sham LV myocytes at all stimulation rates (0.5, 1, 2, and 4 Hz) (n = 6/3; Fig. 3c), which was in good agreement with previous results^24^. These data indicate that enhanced constitutive GIRK activity is required for ventricular repolarization in the failing heart.

Exogenous ACh or vagal stimulation shortens APs of atrial myocytes but not ventricular myocytes in healthy hearts. The physiological basis for the difference in response to ACh has been attributed to the difference in the ACh-induced activation of GIRK currents. Having observed that ACh-induced activation of GIRK currents were also increased in TAC LV myocytes (Fig. 1), we tested whether exogenous ACh can modulate the AP characteristics in these cells. As shown in Fig. 3b, the response to ACh in sham LV myocytes was minimal. ACh had no significant effect on AP morphology and APD_90_ (n = 6/3; Fig. 3d). Resting membrane potential remained unchanged (− 68.9 ± 1.3 mV and − 67.4 ± 1.1 mV before and after ACh, respectively (n = 6/3); *P* > 0.05). Although resting membrane potential was not significantly changed (− 69.6 ± 1.2 mV and − 70.7 ± 1.2 mV before and after ACh, respectively (n = 7/3); *P* > 0.05), a sensitivity to the AP shortening effects of ACh is increased in TAC LV myocytes, as evident by a marked reduction of the APD_90_ (Fig. 3b,d). For example, at a stimulation rate of 1 Hz, the APD_90_ of TAC LV myocytes decreased by 18% after ACh administration, to 20.6 ± 1.8 ms (n = 7/3, *P* < 0.05). Taken together, these data indicate that contribution of GIRK to ventricular repolarization might be enhanced both under baseline condition and during vagal stimulation in TAC mice.

### Constitutive activation of GIRK channels in failing ventricular myocytes is mediated via the M_2_ mAChR

**Figure 4:**
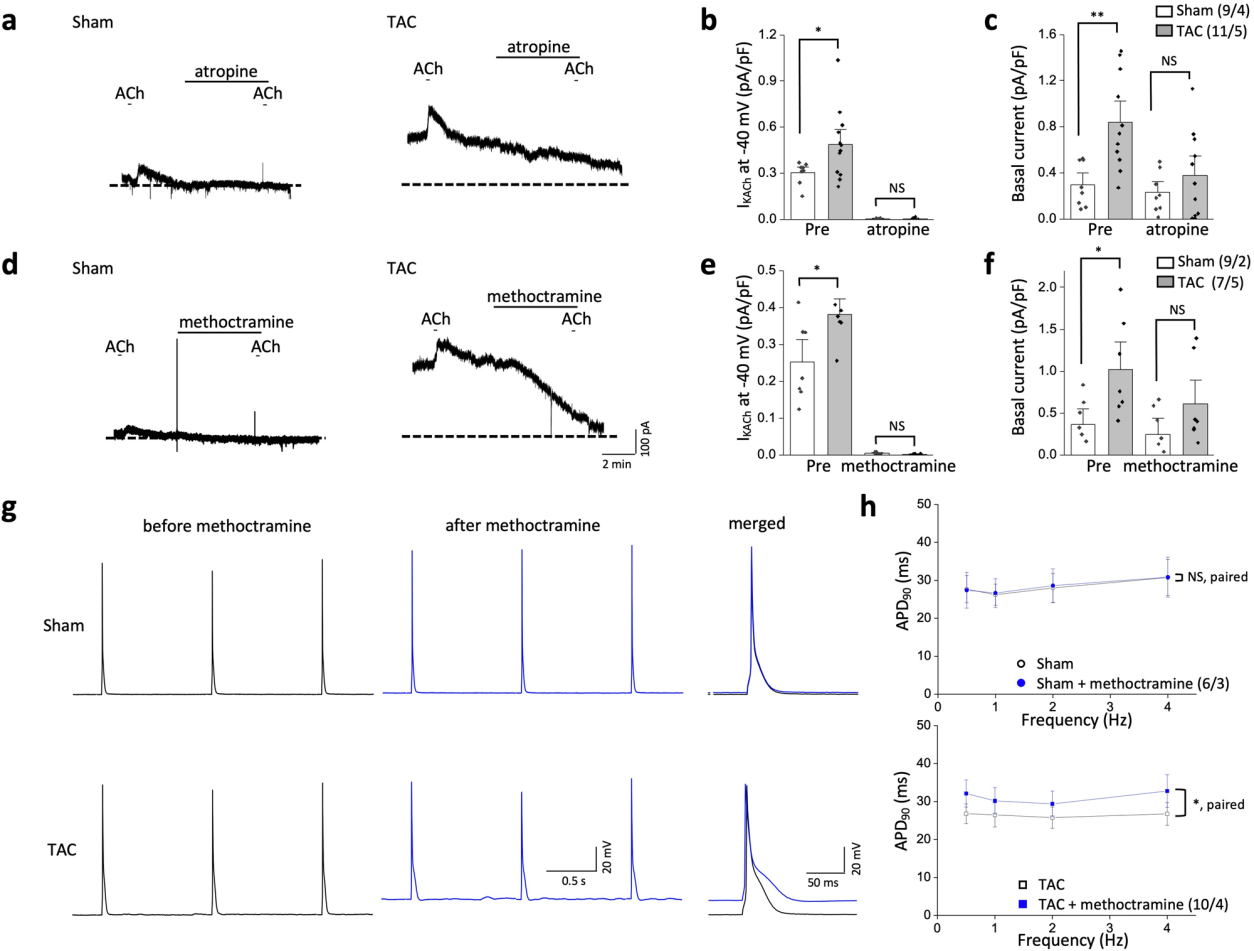
Both constitutive and exogenous acetylcholine (ACh)-induced activation of GIRK channels of transverse aorta constriction (TAC) mice is mediated by M_2_ muscarinic ACh receptors (mAChRs). (**a**) Representative current trance in LV myocytes from sham (left panel) and TAC (right panel) mice exposed to a nonselective muscarinic antagonist, atropine (1 μM) before ACh treatment (10 μM) at a holding potential of − 40 mV for 5 min. (**b**,**c**) Summarized data for the effects of 1 μM atropine on ACh-activated GIRK channels (**b**) and basal current density (**c**) in sham and TAC left ventricular (LV) myocytes. **d** Same as in (**a**), but for the M_2_ mAChR selective antagonist methoctramine. (**e**,**f**) Same as in (**b**,**c**), but for the M_2_ mAChR selective antagonist methoctramine. (**g**) Representative AP traces in LV myocytes stimulated at 1 Hz from sham (upper panel) and TAC (lower panel) before (black) and after (blue) 1 μM methoctramine treatment. Right, superimposed AP traces measured before and after exposure of methoctramine. Methoctramine prolonged action potential duration (APD) only in TAC. (**h**) Summary data of APD at 90% repolarization (APD_90_) in LV myocytes from sham (upper panel) and TAC (lower panel) stimulated at 0.5 Hz, 1 Hz, 2 Hz, and 4 Hz in response to methoctramine. Data represent means ± SEM. The numbers indicate number of myocytes/mice. NS indicates not significantly different. *P < 0.05, **P < 0.01, Student’s paired or unpaired t-test was used as indicated.

We then examined the underlying mechanisms for the enhanced GIRK activity in TAC mice. Cardiac GIRK channels are activated primarily by ACh, a vagal neurotransmitter, through M_2_ mAChR^25^. To examine the role of the mAChR in constitutive GIRK activity in TAC LV myocytes, we first examined the effects of atropine, a nonselective mAChR antagonist on sham and TAC LV myocytes. In both sham and TAC LV myocytes, atropine (1 μM) abolished the exogenous ACh-induced activation of GIRK currents, confirming the role of the muscarinic receptor in exogenous ACh-induced GIRK activation (Fig. 4a,b). In addition, atropine blocked the enhanced basal activity of TAC LV myocytes, abolishing the difference in basal currents between the two groups (sham: 0.2 ± 0.1 pA/pF; TAC, 0.4 ± 0.1 pA/pF; *P* > 0.05; Fig. 4a,c). To further identify the subtype of the mAChRs involved, the effects of a M_2_ mAChR selective inhibitor, methoctramine, were tested. Similar to atropine, methoctramine (1 μM) blocked the basal currents as well as the exogenous ACh-induced GIRK activation and abolished the difference between two groups (sham: 0.2 ± 0.1 pA/pF; TAC, 0.6 ± 0.2 pA/pF; *P* > 0.05; Fig. 4d–f). On the other hand, inhibitors for other mAChR subtypes failed to modify the basal currents of TAC LV myocytes (Supplemental Fig. 3). Thus, constitutive activity of GIRK channels in TAC LV myocytes seems to be dependent of M_2_ mAChRs.

To further probe the role of M_2_ mAChR in constitutively active GIRK currents, we tested the effects of methoctramine on AP characteristics of sham and TAC LV myocytes. Methoctramine treatment (1 μM) produced a marked prolongation of APD at all stimulation rates in TAC LV myocytes without affecting the APs of sham LV myocytes (Fig. 4g–h). APD prolongation in TAC LV myocytes is accompanied by a depolarization in the resting membrane potential (− 70.8 ± 1.3 mV and − 67.9 ± 1.5 mV before and after methoctramine, respectively (n = 10/4); *P* < 0.05). We confirmed that there was no change in the resting membrane potential of sham LV myocytes after treatment with methoctramine (− 68.9 ± 1.8 mV and − 68.7 ± 1.8 mV before and after methoctramine, respectively (n = 6/3); *P* > 0.05). The pattern of APs and the resting membrane potential in response to methoctramine was similar to that in response to tertiapin-Q, further supporting a reduction of constitutive GIRK activities by methoctramine. Together, these data suggest that M_2_ mAChR is involved in the regulation of constitutively active GIRK as well as exogenous ACh-induced GIRK activation in TAC LV myocytes.

### Endogenous ACh is involved in constitutive GIRK activity

**Figure 5:**
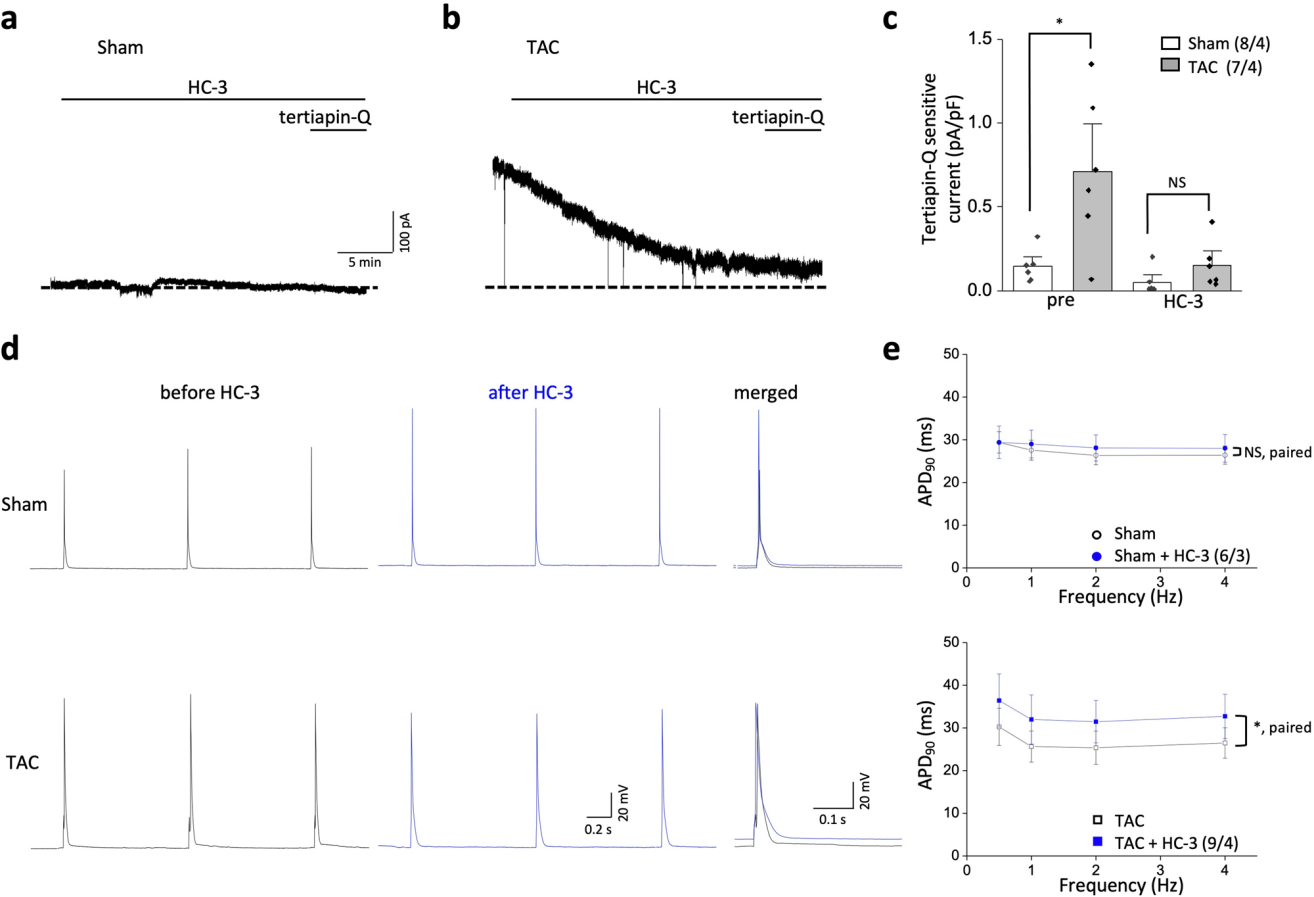
Endogenous acetylcholine (ACh) tonically stimulates GIRK activities in left ventricular (LV) myocytes from transverse aorta constriction (TAC) mice. (**a**,**b**) Representative current trance in LV myocytes from sham (**a**) and TAC (**b**) mice exposed to HC-3 (10 μM) for 24 min at a holding potential of − 40 mV. (**c**) Summarized data for the effects of 10 μM HC-3 on the tertiapinQ-sensitive current density in sham and TAC LV myocytes. Tertiapin Q-sensitive currents were obtained by subtraction. (**d**) Representative action potential (AP) traces in LV myocytes stimulated at 1 Hz from sham (upper panel) and TAC (lower panel) before (black) and after (blue) 10 μM HC-3 treatment. Right, superimposed AP traces measured before and after exposure of HC-3. HC-3 prolonged action potential duration (APD) only in TAC. (**e**) Summary data of APD at 90% repolarization (APD_90_) in LV myocytes from sham (upper panel) and TAC (lower panel) stimulated at 0.5 Hz, 1 Hz, 2 Hz, and 4 Hz in response to HC-3. Data represent means ± SEM. The numbers indicate number of myocytes/mice. NS indicates not significantly different. *P < 0.05, Student’s paired or unpaired t-test was used as indicated.

Cardiomyocytes can secrete significant amounts of ACh via a vesicular ACh transporter (VAChT)-dependent mechanism, similar to that observed in nerve terminals; this cardiomyocyte-derived ACh secretion is increased in heart failure^26,27^. Having observed that the M_2_ mAChR is critical for constitutive GIRK activities of TAC LV myocytes (Fig. 4), we hypothesized that increased ACh secretion from ventricular myocytes could tonically activate M_2_ mAChRs in an autocrine or paracrine manner to generate constitutive GIRK activity in TAC mice. To examine this hypothesis, we depleted endogenous ACh using an inhibitor of ACh synthesis. HC-3 is a specific inhibitor of high-affinity choline transporters, which is a rate-limiting factor for ACh synthesis^28,29^. Previous experiments showed that ACh release from isolated ventricular myocytes was significantly diminished by 30 min treatment of 10 μM HC-3^30^. Application of HC-3 (10 μM, 24 min) completely abolished the difference in tertiapin Q-sensitive GIRK activity between sham and TAC LV myocytes (sham, 0.1 ± 0.03 pA/pF, n = 8/4; TAC, 0.2 ± 0.1 pA/pF, n = 7/4; *P* > 0.05; Fig. 5a–c), indicating that increases in constitutive GIRK activity in TAC LV myocytes might be mediated by endogenous ACh released from ventricular myocytes.

Next, we tested the effects of HC-3 on the AP characteristics of sham and TAC LV myocytes. Consistent with the inhibitory effects of HC-3 on the constitutive GIRK activity, HC-3 produced a marked prolongation of APD at all stimulation rates in TAC LV myocytes, while it had no effects on APs of sham LV myocytes (Fig. 5d,e). APD prolongation of TAC LV myocytes was accompanied by depolarization of the resting membrane potential (− 68.6 ± 1.1 mV and − 64.6 ± 1.6 mV before and after HC-3, respectively (n = 9/4); *P* < 0.05). The resting membrane potential of sham LV myocytes remained unchanged (− 69.9 ± 1.0 mV and − 68.6 ± 1.9 mV before and after HC-3, respectively (n = 6/3); *P* > 0.05). These data further support the role of endogenous ACh in constitutive GIRK activity. Taken together, these data suggest that constitutive GIRK activity might be produced by endogenously released ACh through tonic activation of M_2_ mAChR.

### Inhibition of constitutive GIRK activity leads to QT prolongation and spontaneous arrythmia in TAC mice

**Figure 6:**
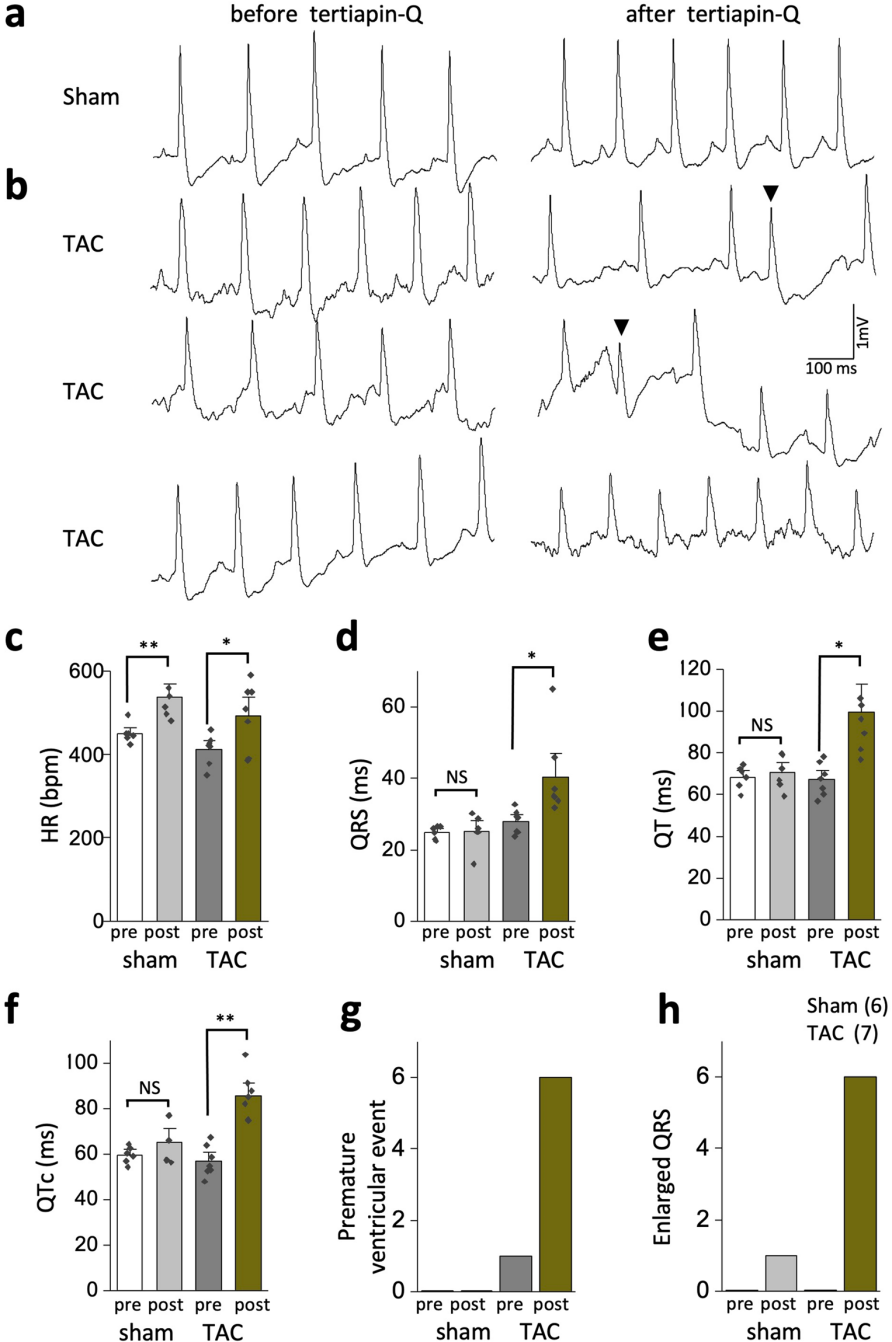
Inhibition of GIRK activity with tertiapin-Q induces QT prolongation and arrhythmia in transverse aorta constriction (TAC) mice. (**a**,**b**) Representative lead-II electrocardiogram (ECG) traces from sham (**a**) and TAC (**b**) mice. Mice were anesthetized using isoflurane, and the basal ECG was monitored for 10–15 min. The ECG was further monitored for 15 min following an intraperitoneal injection of tertiapin-Q (5 mg/kg). The trace from sham mice indicates sinus rhythm. ECGs from TAC mice show premature ventricular event (arrowhead, 1st and 2nd traces) and a episode of ventricular tachycardia (3rd trace) after tertiapin-Q treatment. (**c**–**h**) Summarized data for heart rate (HR) (**c**), QRS (**d**), QT (**e**), QTc (**f**), premature ventricular event (**g**), and incidence of the enlarged QRS complex (**h**). n = 6 in the sham group and n = 7 in the TAC group. NS indicates not significantly different. *P < 0.05, **P < 0.01, Student’s t-test.

Delayed repolarization is known to underlie increased arrhythmogenesis in heart failure^31^. Having observed that inhibition of constitutively active GIRK induced delayed repolarization in TAC LV myocytes (Fig. 3), we examined whether inhibition of constitutively active GIRK might increase the arrhythmic risk in TAC mice. For this, we tested the effects of tertiapin-Q on ECG parameters in isoflurane-anesthetized mice. Consistent with previous reports^32^, intraperitoneal injection of tertiapin-Q (5 mg/kg) increased the heart rate without affecting the QRS, QT, and QTc intervals in sham mice (Fig. 6a,c–f). In contrast, tertiapin-Q not only increased the heart rate, but also induced excessive prolongation of the QT and QTc interval in TAC mice compared with sham mice at a similar heart rate (Fig. 6b,c–f). QT was increased from 67.4 ± 3.0 ms to 99.8 ± 8.6 ms (n = 7, *P* < 0.05) and QTc was increased from 56.9 ± 2.6 ms to 85.8 ± 3.7 ms (n = 7, *P* < 0.01) in TAC mice compared with minimal prolongation in sham animals (QT: from 68.4 ± 2.3 ms to 70.5 ± 3.3 ms (n = 6, *P* > 0.05); QTc: from 59.7 ± 1.5 ms to 65.2 ± 4.0 ms (n = 6, *P* > 0.05). We detected an increased frequency of premature ventricular contractions (PVCs) in TAC mice after tertiapin-Q but not in sham mice (Fig. 6b,g). Spontaneous self-terminating episodes of tachyarrhythmias characterized by fusions beats, wider QRS complexes with a different morphology, and atrio-ventricular dissociation were also detected in six of the seven TAC mice after tertiapin-Q treatment, but in only one of the six sham mice (Fig. 6b,h). These data suggest that inhibition of constitutively active GIRK increased arrhythmia development in TAC mice, implying a protective role of constitutively active GIRK channels against the electrical instability and arrythmia in heart failure.

## Discussion

In healthy hearts, GIRK channels are activated in response to ACh release from parasympathetic nerve terminals and their function is known to be restricted to the atrial and nodal cells. Using TAC mice, a useful model of heart failure with lethal arrhythmias, our work reveals that constitutive GIRK activity is induced in the failing ventricular myocytes (Figs. 1, 2). Blocking constitutive GIRKs with its specific inhibitor, tertiapin-Q, results in APD prolongation in failing ventricular myocytes but not in control cells, indicating that constitutive GIRK activity protects the failing heart from APD prolongation (Fig. 3). We demonstrated that this constitutive GIRK activity is mediated possibly by tonic activation of M_2_ mAChRs via endogenously released ACh from TAC LV myocytes (Figs. 4, 5). While long-term exposure to a M_2_ mAChR agonist has been shown to decrease GIRK currents by desensitization of the GPCRs^33^, it seems that the constitutive M_2_ mAChR/GIRK activity does not produce the same effect, so those M_2_ mAChRs are still able to mediate APD shortening when a high concentration of ACh is applied (Figs. 1, 3). To clarify the contribution made by constitutive GIRK activity to the arrhythmia prevention, we tested the effects of tertiapin-Q on arrhythmicity. Tertiapin-Q significantly increased the QT interval and spontaneous ventricular arrhythmias in TAC mice while having no significant effect in sham mice, most likely by prolonging the APD (Fig. 6). Collectively, our findings provide the first evidence that constitutive activity of GIRK channels contributes to arrhythmia prevention in failing hearts.

It is well known that APD prolongation underlies lethal ventricular arrhythmias in heart failure^34,35^. Recent data from experimental models of cardiac hypertrophy or failure, and terminal heart failure in humans, indicate that the prolongation is primarily due to changes in repolarizing K^+^ currents^22^. Down-regulation of multiple K^+^ currents may cause abnormalities in repolarization and contribute to the APD prolongation. In addition to a reduction in K^+^ currents, remodeling in heart failure also causes increases in the depolarizing currents such as voltage-gated Na^+^ currents and voltage-gated Ca^2+^ currents. This further prolongs repolarization and the APD, leading to arrhythmogenesis^36^. For K^+^ channels, the most consistent change is a reduction in the phase 1 repolarizing transient outward potassium current (I_to_)^13,37^. In a mouse model of heart failure, reductions of I_to_ in ventricular myocytes is primarily due to reduction in a pore-forming α-subunit Kv4.2 and less so for Kv4.3 α-subunit^38^ while reduced expression of the associated β-subunit KChIP2 has been reported to be responsible for the reduction in I_to_ noted in human heart failure and in a mouse model of hypertrophy^39–41^. Another denominator of heart failure remodeling is a reduction in the phase 3 repolarizing I_K1_, although findings are not as consistent as observed for I_to_^13,42–44^. The main strategy for antiarrhythmic drug development in recent decades has been to identify the culprit ion channels responsible for arrhythmias and develop specific ion channel blockers (or activators) to target individual channels. However, clinical trials of ion channel blockers have met unexpected failures, as summarized by Sanderson^45^ in the Editorial on SWORD and CAST II trials: “In few specialties of medicine are new promising drugs shown to be so much inferior to placebo and, even worse, to increase mortality.” Our study found that tertiapin-Q induced APD prolongation and increased the PVCs, suggesting that because of downregulation of multiple K^+^ currents, constitutively active GIRKs may function as the repolarization reserve in the failing ventricles. This is consistent with the inward rectification property of GIRK channels. Similar to Kir2.x channels conducting I_K1_, albeit with less rectification, they can conduct repolarizing current, preventing excessive AP prolongation^22^. In this study, we demonstrated that both exogenous ACh-induced activation of GIRK currents and constitutively active GIRK currents in TAC ventricular myocytes. The currents density of ACh-induced GIRK currents was about 0.6 pA/pF at − 40 mV (Figs. 1, 2). Several studies have provided evidence that a similar increase of GIRK currents has great effects on APDs of cardiac myocytes. For exmpale, a study of adenosine-induced GIRK currents in heart failure dogs showed that about 0.5 pA/pF of GIRK currents at − 50 mV has significant effects on action potenatials of cardiac myocytes^46^. The current density of constitutively active GIRK currents in TAC ventricular myocytes is about 0.4 pA/pF (tertiapin-Q-sensitive basal currents; Fig. 2). Constitutive activity of GIRK channels can contribute to low resting membrane potential as well as a repolarizing current. Although it is small, since it is non-inactivating the amount of charge that can be carried by it over the time course of an AP might be large enough to produce a major impact on APDs. Many studies have shown that small, but persistent ionic currents have a major impact on ventricular APs^47,48^. For example, approximately 0.1 pA/pF increase in persistent sodium currents in mice ventricular myocytes contribute to APD prolongation and QT interval prolongation^49^. Therefore, our findings suggest that the modulation of ventricular GIRK activity is a potentially useful approach to preventing ventricular APD prolongation in patients with heart failure.

The constitutive component of the GIRK channel is tiny in healthy hearts, and GIRK activation normally requires an increased parasympathetic or vagal tone. An induction of constitutive GIRK activity has been reported in conjunction with atrial fibrillation^14,15^. Thus, constitutive GIRK activity is another example of opposite consequences of remodeling in atria and ventricles, since they are proarrhythmic in the failing atria^14^. This might occur because the hallmark of atrial fibrillation is shortened and triangulated atrial APs, whereas the characteristic feature of ventricular remodeling in heart failure is a prolonged ventricular AP^22^. Further studies in other animal models of cardiac disease and in humans with heart failure are needed to explore this possibility.

Recently, a non-neuronal cholinergic system has been recognized to exert beneficial effects in cardiovascular disease, such as in sympathetic hyperactivity-induced cardiac remodeling and dysfunction as well as myocardial infarction^26^. The existence of the cardiac non-neuronal cholinergic system has been confirmed by the presence of cholinergic markers in the cardiomyocytes, which are crucial for synthesis (choline acetyltransferase, ChAT), storage (VAChT), reuptake of choline for synthesis (high-affinity choline transporter, CHT1), and degradation (acetylcholinesterase, AChE) of ACh^26^. Additionally, the non-neuronal ACh derived from cardiomyocytes was detected intracellularly and extracellularly in the presence of AChE inhibitors such as donepezil, physostigmine, and pyridostigmine^50–52^. The non-neuronal ACh released from cardiomyocytes can activate mAChR in an auto/paracrine manner to regulate the hypertrophic signals or cardiac energy metabolism^26^. However, it has not been investigated whether this non-neuronal cholinergic system would regulate the electrical activity in cardiac diseases. Our data showed that non-neuronal ACh released from cardiomyocytes contributes to the stability of the cardiac electrical activity as inhibition of M_2_ mAChR and choline uptake by methoctramine and HC-3, respectively, blunted the constitutive GIRK channels and their antiarrhythmic effects in the failing ventricular myocytes. However, we cannot rule out that the alterations within the M_2_ mAChR/G protein/GIRK channel complex might also contribute to constitutive GIRK activity in these cells. Nevertheless, the non-neuronal cholinergic system exerts cardioprotective effects via the activation of GIRK channels in ventricular myocytes, providing novel insights into the understanding of the cellular mechanisms underlying the cardioprotective effects of non-neuronal cholinergic system. Previously it has been shown that atropine can induce ventricular tachycardia in patients with ischemic heart diseases but rarely in normal subjects^53–55^. Although the role of ventricular GIRK channels in heart diseases have been insufficiently investigated, the susceptibility to ventricular arrhythmias through atropine may result from enhanced GIRK channel function in these patients.

In this study we attempted to examine a role of constitutive GIRK currents in modulating ventricular electrical activity. Although these data are interesting, they may not be extrapolated to the diseased human myocardium because of the presence of comorbid conditions and marked differences in AP profiles. AP repolarization and morphology vary greatly between regions of the ventricle due to region- and species-dependent differences in channel expression across the ventricle wall^56^. Since the present study indiscriminately used ventricular myocytes across ventricle walls, it remains to be determined whether GIRK currents in TAC mice would be heterogenous in the ventricles and whether this might affect the diversity in APD. Another limitation is the concern that ECG recordings were performed under anesthesia. General anesthesia minimizes the stress and ensures minimal movement of the animal. However, the effect of anesthesia on the heart rate must be considered for data interpretation^57^.

In conclusion, our data suggest that heart failure increases constitutive GIRK activity in ventricular myocytes which, in turn, prevents rhythm disturbances, likely by maintaining ventricular repolarization. This neuronal control-independent, constitutive activity of GIRK channels in heart failure may be of considerable clinical relevance because it could serve as a therapeutic target for maintaining ventricular function and reducing arrhythmias in heart diseases.

## Methods

### Ethics statement

All animal experiments were conducted in accordance with the guidelines of the IACUC at Sungkyunkwan University School of Medicine (approval no. SKKUIACUC2019-07-11-3) and comply with the National Institutes of Health guide for the care and use of laboratory animals (NIH Publications No. 8023, 8th edition, 2011). Sungkyunkwan University School of Medicine is an Association for Assessment and Accreditation of Laboratory Animal Care International (AAALAC International) accredited facility and abide by the Institute of Laboratory Animal Resources (ILAR) guide. This manuscript was written in accordance with the ARRIVE guidelines (Animal Research: Reporting of In Vivo Experiments).

### Animals

The animal experiments in this study were performed with C57BL/6N mice. Adult mice (purchased from Orient Bio Inc., Seongnam, Korea) were housed in the specific pathogen-free level animal facility at Sungkyunkwan university school of medicine and maintained in an enriched environment with a temperature-controlled room in a 12 h light–dark cycle, with food and water available. Mice were injected with heparin (1.0 units/kg) and euthanized using carbon dioxide (CO_2_), followed by cardiac excision.

### Transverse aortic constriction

TAC was performed on 8-week-old male mice as described earlier^58^. Mice were anesthetized with 3% isoflurane mixed with 0.5–1.0 L/min 100% O_2_ and were fixed in a supine position on the heating pad with body temperature 37 °C. Endotracheal intubation was rapidly performed and connected to a ventilator (SAR 1000(12-03100), L.M.S. KOREA). A midline incision was made at the upper sternum and, the transverse aortic arch was isolated in the middle of the arch between the innominate and left common carotid arteries. A 7–0 nylon suture ligature was tied around the transverse aorta against a 27-gauge needle with 0.4 mm OD, and then the needle was quickly removed. The chest and skin were closed. After spontaneous breathing appeared, mice were removed from intubation and maintained at 37 °C until recovery. Sham-operated mice were subjected to the same surgical procedure without aortic constriction. Following TAC, the peak trans-TAC pressure gradient was determined by application of Color Doppler imaging to the aortic arch, distal to the TAC constriction, as previously described^59–61^. Color Doppler imaging revealed either laminar blood flow in sham-operated mice, or turbulent blood flow in TAC mice. A peak pressure TAC gradient was calculated using the modified Bernoulli equation (Pressure gradient = 4 * velocity^2^)^59–61^.

### Echocardiography

Cardiac function was monitored during high-resolution transthoracic echocardiography (TTE) with Vevo 2100 Ultrasound System designed for small animals (Fujifilm Visual Sonics Inc., Toronto, Ontario, Canada). A MS-400 high-frequency ultrasound probe operating at 40 MHz was used. M-mode echocardiography was performed after 3 weeks of surgery and keep monitoring every two weeks. After the chest was shaved, mice were anesthetized under 1–2% isoflurane mixed with 0.5–1.0 L/min 100% O_2_, fixed in a supine position on the heating pad with body temperature 37 °C. On the shaven chest, with the warm echo gel applied, the heart was scanned in the two-dimensional mode in the parasternal long axis view by positioning the transducer along the long axis of the LV. A 2D guided M-mode echocardiogram was obtained at a sweep speed of 200 mm/s. The left ventricles (LV) end-diastolic and end-systolic dimensions (LVID;d and LVID;s), interventricular septum thickness, and posterior wall thickness were measured. The LV percent fractional shortening (FS%) and LV ejection fraction (EF%) was calculated using the equation FS% = [(LVID;d − LVID;s)/LVID;d] × 100, EF% = [(LVV;d − LVV;s)/LVV;d] × 100 (LVV;d LV volume; end diastolic and end systolic).

### Electrocardiography (ECG)

Surface ECG signal (lead II via limb electrodes) was recorded during high-resolution transthoracic echocardiography (TTE) with Vevo 2100 Ultrasound System designed for small animals (Fujifilm Visual Sonics Inc., Toronto, Ontario, Canada). The mice were anesthetized with 1–2% isoflurane mixed with 0.5–1.0 L/min 100% O_2_, and kept in supine position on a heated pad in order to maintain their body temperature at 37 °C. The mice's paws were attached to the platform's electrode pads using ECG gel and secured with skin tape in order to record the ECG signal. Additionally, heart rate and respiration were monitored via the ECG pads. The whole recorded ECG tracing in the present study was visually reviewed for detecting possible arrhythmias or other aberrant ECG complexes. Heart rate and QRS interval duration were determined by averaging three consecutive beats during sinus rhythm as previously described^62,63^. Defined timepoints were onset and offset of the P-wave, Q-wave onset, and R- and S-wave peaks and offset of the QRS, and T wave. By using these timepoints P-wave duration, PQ interval, Q wave duration, QRS width, and QT interval were estimated. In addition, amplitudes of the P, Q, R, and S waves were defined using onset of the P wave as isoelectric line. Heart rate-related QT intervals (QTc) were corrected by using the Bazett formula^64,65^.

### Isolation of mouse ventricular myocytes

Adult ventricular myocytes were isolated from mice 11 weeks after TAC or sham operation using enzymatic dissociation as previously described^19^. Briefly, the heart was cannulated and retrogradely perfused via the aorta with a Ca^2+^-free normal Tyrode solution containing collagenase (1 mg/ml, Worthington, type 2) on a Langendorff column at 37 °C. Isolated cardiac myocytes were kept in high K^+^, low Cl^–^ solution at 4 °C until use. Normal Tyrode solution contained (mM): 140 NaCl, 5.4 KCl, 0.5 MgCl_2_, 1.8 CaCl_2_, 10 glucose, and 5 Hepes, titrated to pH 7.4 with NaOH. The Ca^2+^-free solution contained (mM): 140 NaCl, 5.4 KCl, 0.5 MgCl_2_, 10 glucose, and 5 Hepes, titrated to pH 7.4 with NaOH. The high K^+^, low Cl^–^ solution contained (mM): 70 KOH, 40 KCl, 50 L-glutamic acid, 20 taurine, 20 KH_2_PO_4_, 3 MgCl_2_, 10 glucose, 10 Hepes, and 0.5 EGTA. Only Ca^2+^-tolerant, rod-shaped myocytes with cross-striations and without spontaneous contractions or significant granulation were selected for electrophysiological experiments.

### Whole-cell patch-clamp recording

The perforated patch clamp technique was used to record membrane currents or voltages from single isolated myocytes using nystatin (200 μg/ml, MP Biomedicals) with an EPC-10 patch-clamp amplifier (HEKA Instrument, Germany) as described previously^62,63,66^. In current‐clamp mode, APs were evoked by a brief suprathreshold current pulse. In voltage‐ clamp mode, access resistance was monitored through the experiments, and data were accepted only when access resistance was kept at < 10 MΩ. Filtered signals (10 kHz) from a patch‐clamp amplifier were digitized at 20 kHz and stored on a personal computer for later analysis. The patch pipettes (World Precision Instruments) were made by a Narishige puller (PP‐830; Narishige) and had a resistance of 3 ± 0.5 MΩ when filled with the pipette solution. All electrophysiological experiments were performed at 34–35 °C. The bath solution (or normal Tyrode solution) contained (mM): 140 NaCl, 5.4 KCl, 0.5 MgCl_2_, 1.8 CaCl_2_, 10 glucose, and 5 Hepes, titrated to pH 7.4 with NaOH. The pipette solution for perforated patches contained (mM): 140 KCl, 10 Hepes, 1 MgCl_2_, and 5 EGTA, titrated to pH 7.2 with KOH. Data were recorded and collected by using Patch-Master software.

### Chemicals and reagents

Tertiapin-Q and PD-102807 were purchased from Tocris Bioscience (Bristol); All other drugs and chemicals were purchased from Sigma‐Aldrich.

### Statistics

Data were analyzed with Origin (Version 6.1; OriginLab). All results are presented as the mean ± SEM with the number of cells and mice used in each experiment. Statistical significance was evaluated using the Student’s t-test or ANOVA Tukey tests were performed after normal distribution of data was examined with Shapiro–Wilk test. The level of significance was indicated by the number of marks. *P* > 0.05 was regarded as not significantly different.

### Supplementary Information

Supplementary Figures.

## Data Availability

All study data are included in the article and/or its supplementary files.

## References

[1] Lüscher C, Slesinger PA (2010). Emerging roles for G protein-gated inwardly rectifying potassium (GIRK) channels in health and disease. Nat. Rev. Neurosci..

[2] Sakmann B, Noma A, Trautwein W (1983). Acetylcholine activation of single muscarinic K^+^ channels in isolated pacemaker cells of the mammalian heart. Nature.

[3] Dhein S, van Koppen CJ, Brodde OE (2001). Muscarinic receptors in the mammalian heart. Pharmacol. Res..

[4] Wickman K, Nemec J, Gendler SJ, Clapham DE (1998). Abnormal heart rate regulation in GIRK4 knockout mice. Neuron.

[5] Bettahi I, Marker CL, Roman MI, Wickman K (2002). Contribution of the Kir3.1 subunit to the muscarinic-gated atrial potassium channel IKACh. J. Biol. Chem..

[6] Mesirca P (2013). The G-protein-gated K^+^ channel, IKACh, is required for regulation of pacemaker activity and recovery of resting heart rate after sympathetic stimulation. J. Gen. Physiol..

[7] Lee SW (2018). Atrial GIRK channels mediate the effects of vagus nerve stimulation on heart rate dynamics and arrhythmogenesis. Front. Physiol..

[8] Dobrzynski H, Janvier NC, Leach R, Findlay JB, Boyett MR (2002). Effects of ACh and adenosine mediated by Kir3.1 and Kir3.4 on ferret ventricular cells. Am. J. Physiol. Heart Circ. Physiol..

[9] Dobrzynski H (2001). Distribution of the muscarinic K^+^ channel proteins Kir3.1 and Kir3.4 in the ventricle, atrium, and sinoatrial node of heart. J. Histochem. Cytochem..

[10] Riehle C, Bauersachs J (2019). Small animal models of heart failure. Cardiovasc. Res..

[11] Kuwabara Y (2013). Increased expression of HCN channels in the ventricular myocardium contributes to enhanced arrhythmicity in mouse failing hearts. J. Am. Heart Assoc..

[12] Akar FG, Tomaselli GF (2005). Ion channels as novel therapeutic targets in heart failure. Ann. Med..

[13] Nattel S, Maguy A, Le Bouter S, Yeh YH (2007). Arrhythmogenic ion-channel remodeling in the heart: Heart failure, myocardial infarction, and atrial fibrillation. Physiol. Rev..

[14] Dobrev D (2005). The G protein-gated potassium current I(K, ACh) is constitutively active in patients with chronic atrial fibrillation. Circulation.

[15] Voigt N (2008). Changes in I K, ACh single-channel activity with atrial tachycardia remodelling in canine atrial cardiomyocytes. Cardiovasc. Res..

[16] Voigt N, Abu-Taha I, Heijman J, Dobrev D (2014). Constitutive activity of the acetylcholine-activated potassium current IK,ACh in cardiomyocytes. Adv. Pharmacol..

[17] Djebari S (2021). G-protein-gated inwardly rectifying potassium (Kir3/GIRK) channels govern synaptic plasticity that supports hippocampal-dependent cognitive functions in male mice. J. Neurosci..

[18] Gonzalez JC, Epps SA, Markwardt SJ, Wadiche JI, Overstreet-Wadiche L (2018). Constitutive and synaptic activation of GIRK channels differentiates mature and newborn dentate granule cells. J. Neurosci..

[19] Cho H, Nam GB, Lee SH, Earm YE, Ho WK (2001). Phosphatidylinositol 4,5-bisphosphate is acting as a signal molecule in alpha(1)-adrenergic pathway via the modulation of acetylcholine-activated K(+) channels in mouse atrial myocytes. J. Biol. Chem..

[20] Jin W, Lu Z (1998). A novel high-affinity inhibitor for inward-rectifier K^+^ channels. Biochemistry.

[21] Calloe K, Goodrow R, Olesen SP, Antzelevitch C, Cordeiro JM (2013). Tissue-specific effects of acetylcholine in the canine heart. Am. J. Physiol. Heart Circ. Physiol..

[22] Schmitt N, Grunnet M, Olesen SP (2014). Cardiac potassium channel subtypes: New roles in repolarization and arrhythmia. Physiol. Rev..

[23] Zhao Y, Gameiro-Ros I, Glaaser IW, Slesinger PA (2021). Advances in targeting GIRK channels in disease. Trends Pharmacol. Sci..

[24] Cha TJ (2006). Kir3-based inward rectifier potassium current: Potential role in atrial tachycardia remodeling effects on atrial repolarization and arrhythmias. Circulation.

[25] Kurachi Y, Ishii M (2004). Cell signal control of the G protein-gated potassium channel and its subcellular localization. J. Physiol..

[26] Saw EL, Kakinuma Y, Fronius M, Katare R (2018). The non-neuronal cholinergic system in the heart: A comprehensive review. J. Mol. Cell. Cardiol..

[27] Roy A (2016). Cardiac acetylcholine inhibits ventricular remodeling and dysfunction under pathologic conditions. FASEB J..

[28] Okuda T, Haga T (2000). Functional characterization of the human high-affinity choline transporter. FEBS Lett..

[29] Okuda T (2000). Identification and characterization of the high-affinity choline transporter. Nat. Neurosci..

[30] Rocha-Resende C (2012). Non-neuronal cholinergic machinery present in cardiomyocytes offsets hypertrophic signals. J. Mol. Cell Cardiol..

[31] Tomaselli GF, Zipes DP (2004). What causes sudden death in heart failure?. Circ. Res..

[32] Hashimoto N, Yamashita T, Tsuruzoe N (2006). Tertiapin, a selective IKACh blocker, terminates atrial fibrillation with selective atrial effective refractory period prolongation. Pharmacol. Res..

[33] Yamanushi TT (2007). Role of internalization of M2 muscarinic receptor via clathrin-coated vesicles in desensitization of the muscarinic K^+^ current in heart. Am. J. Physiol. Heart Circ. Physiol..

[34] Coronel R (2013). Electrophysiological changes in heart failure and their implications for arrhythmogenesis. Biochim. Biophys. Acta.

[35] Li GR, Lau CP, Leung TK, Nattel S (2004). Ionic current abnormalities associated with prolonged action potentials in cardiomyocytes from diseased human right ventricles. Heart Rhythm.

[36] Rahm AK, Lugenbiel P, Schweizer PA, Katus HA, Thomas D (2018). Role of ion channels in heart failure and channelopathies. Biophys. Rev..

[37] He Q, Feng Y, Wang Y (2015). Transient outward potassium channel: A heart failure mediator. Heart Fail. Rev..

[38] Petkova-Kirova PS (2006). Electrical remodeling of cardiac myocytes from mice with heart failure due to the overexpression of tumor necrosis factor-alpha. Am. J. Physiol. Heart Circ. Physiol..

[39] Kuo HC (2001). A defect in the Kv channel-interacting protein 2 (KChIP2) gene leads to a complete loss of I(to) and confers susceptibility to ventricular tachycardia. Cell.

[40] Radicke S (2006). Functional modulation of the transient outward current Ito by KCNE beta-subunits and regional distribution in human non-failing and failing hearts. Cardiovasc. Res..

[41] Grubb S, Calloe K, Thomsen MB (2012). Impact of KChIP2 on cardiac electrophysiology and the progression of heart failure. Front. Physiol..

[42] Rozanski GJ, Xu Z, Whitney RT, Murakami H, Zucker IH (1997). Electrophysiology of rabbit ventricular myocytes following sustained rapid ventricular pacing. J. Mol. Cell Cardiol..

[43] Soltysinska E (2009). Transmural expression of ion channels and transporters in human nondiseased and end-stage failing hearts. Pflugers Arch..

[44] Tsuji Y (2000). Pacing-induced heart failure causes a reduction of delayed rectifier potassium currents along with decreases in calcium and transient outward currents in rabbit ventricle. Cardiovasc. Res..

[45] Sanderson J (1996). The SWORD of damocles. Lancet.

[46] Long VP (2020). Chronic heart failure increases negative chronotropic effects of adenosine in canine sinoatrial cells via A1R stimulation and GIRK-mediated I(Kado). Life Sci..

[47] Saint DA (2008). The cardiac persistent sodium current: An appealing therapeutic target?. Br. J. Pharmacol..

[48] Belardinelli L, Giles WR, Rajamani S, Karagueuzian HS, Shryock JC (2015). Cardiac late Na^+^ current: Proarrhythmic effects, roles in long QT syndromes, and pathological relationship to CaMKII and oxidative stress. Heart Rhythm.

[49] Lu Z (2013). Increased persistent sodium current due to decreased PI3K signaling contributes to QT prolongation in the diabetic heart. Diabetes.

[50] Kakinuma Y, Akiyama T, Sato T (2009). Cholinoceptive and cholinergic properties of cardiomyocytes involving an amplification mechanism for vagal efferent effects in sparsely innervated ventricular myocardium. FEBS J..

[51] Rana OR (2010). Acetylcholine as an age-dependent non-neuronal source in the heart. Auton. Neurosci..

[52] Roy A (2013). Cardiomyocyte-secreted acetylcholine is required for maintenance of homeostasis in the heart. FASEB J..

[53] Massumi RA (1972). Ventricular fibrillation and tachycardia after intravenous atropine for treatment of bradycardias. N. Engl. J. Med..

[54] Goel VK, Mehrotra TN, Gupta SK (1981). Ventricular tachyarrhythmia: Complication of atropine therapy in acute myocardial infarction. Indian Heart J..

[55] Magnano AR, Holleran S, Ramakrishnan R, Reiffel JA, Bloomfield DM (2002). Autonomic nervous system influences on QT interval in normal subjects. J. Am. Coll. Cardiol..

[56] McKinnon D, Rosati B (2016). Transmural gradients in ion channel and auxiliary subunit expression. Prog. Biophys. Mol. Biol..

[57] Stracina T, Ronzhina M, Redina R, Novakova M (2022). Golden standard or obsolete method? Review of ECG applications in clinical and experimental context. Front. Physiol..

[58] Rockman HA (1991). Segregation of atrial-specific and inducible expression of an atrial natriuretic factor transgene in an in vivo murine model of cardiac hypertrophy. Proc. Natl. Acad. Sci. U S A.

[59] Mohammed SF (2012). Variable phenotype in murine transverse aortic constriction. Cardiovasc. Pathol..

[60] Richards DA (2019). Distinct phenotypes induced by three degrees of transverse aortic constriction in mice. Sci. Rep..

[61] Li L (2016). Assessment of cardiac morphological and functional changes in mouse model of transverse aortic constriction by echocardiographic imaging. J. Vis. Exp..

[62] Pyun JH (2018). Cardiac specific PRMT1 ablation causes heart failure through CaMKII dysregulation. Nat. Commun..

[63] Jeong MH (2017). Cdon deficiency causes cardiac remodeling through hyperactivation of WNT/β-catenin signaling. Proc. Natl. Acad. Sci. U S A.

[64] Liu JF (2011). Risk factors for recurrent syncope and subsequent fatal or near-fatal events in children and adolescents with long QT syndrome. J. Am. Coll. Cardiol..

[65] Ishikawa J, Ishikawa S, Kario K (2015). Prolonged corrected QT interval is predictive of future stroke events even in subjects without ECG-diagnosed left ventricular hypertrophy. Hypertension.

[66] Kim JG (2016). Impaired inactivation of L-Type Ca^2+^ current as a potential mechanism for variable arrhythmogenic liability of HERG K^+^ channel blocking drugs. PLoS ONE.

