# Supplementary material for: Increased GIRK channel activity prevents arrhythmia in mice with heart failure by enhancing ventricular repolarization
Source: Sci Rep. 2023 Dec 18;13:22479. doi: 10.1038/s41598-023-50088-2 (PMC10728207; doi:10.1038/s41598-023-50088-2)
Supplement: Supplementary file 1 — Supplementary Figures. [file 41598_2023_50088_MOESM1_ESM.docx]

**Supplemental Figures**

**
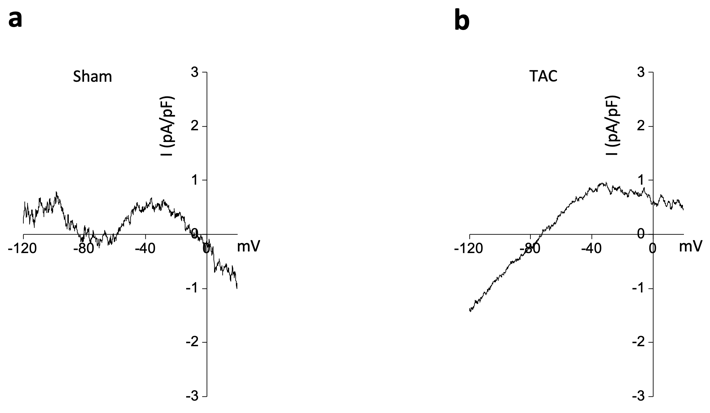
**

**Supplemental Figure 1. Current-voltage relationships of ACh-sensitive current components in sham and transverse aorta constriction (TAC) LV myocytes.** Current-voltage relationships of ACh-sensitive current components in sham ventricular myocytes (a) and TAC ventricular myocytes (b). Data were calculated from Fig. 1g.

**
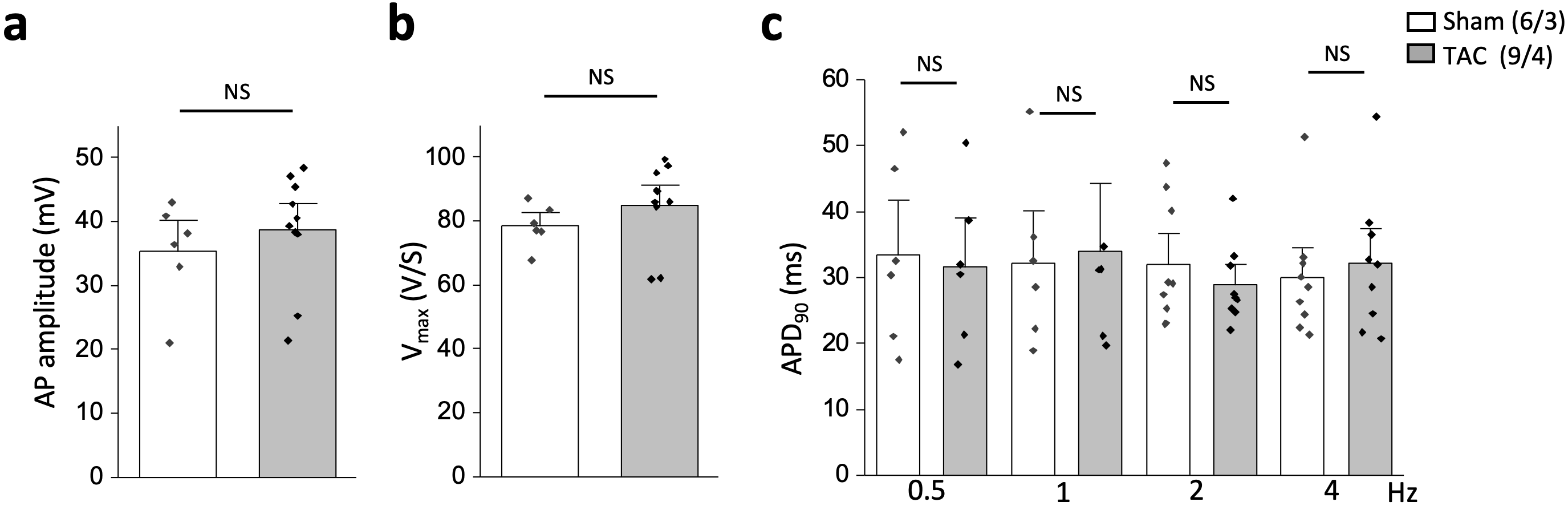
**

**Supplemental Figure 2. Action potential (AP) parameters of left ventricular (LV) myocytes from sham and transverse aorta constriction (TAC) mice.** Summarized data for AP amplitude (**a**), AP upstroke velocity (V_max_; **b**), and AP duration at 90% repolarization (APD_90_; **c**) in LV myocytes from sham and TAC mice stimulated at 0.5 Hz, 1 Hz, 2 Hz, and 4 Hz. The numbers indicate number of myocytes/mice. NS indicates not significantly different, Student’s t-test.


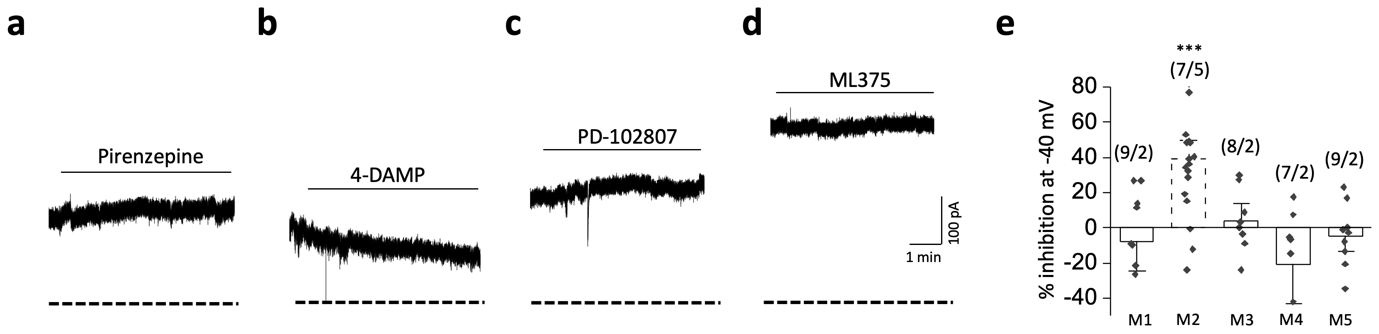


**Supplemental Figure 3. Effects of selective antagonists for the muscarinic acetylcholine receptor (mAChR) subtypes on the basal currents in failing ventricular myocytes.** **a**-**d** Selective antagonist for M1 (pirenzepine, 1 μM; a), M3 (4-DAMP, 10 μM; b), M4 (PD-102807, 200 nM; c), or M5 (ML375, 10 μM; d) was applied to left ventricular myocytes from transverse aorta constriction (TAC) mice at a holding potential of −40 mV for 5 min. **e** Extent of basal current inhibition at −40 mV summarized from (a)-(d). The numbers indicate number of myocytes/mice. The extent of methoctramine-induced basal current inhibition was redrawn from Fig. 4f for comparison (dashed line). ****P < 0.001, ANOVA Tukey test.
